# Rural Households’ Poverty and Relocation and Settlement: Evidence from Western China

**DOI:** 10.3390/ijerph16142609

**Published:** 2019-07-22

**Authors:** Wei Liu, Jie Xu, Jie Li, Shuzhuo Li

**Affiliations:** 1 Northwest Center for Rural Vitalization Research, School of Public Administration, Xi’an University of Architecture and Technology, Xi’an 710055, China; 2 School of Public Policy and Administration, Xi’an Jiaotong University, Xi’an 710049, China

**Keywords:** relocation and settlement, voluntary poverty, transient poverty, chronic poverty, rural household

## Abstract

Based on survey data collected from five counties across southern Shaanxi, China, the present study employs a multinomial logistic model to explore the main factors related to the type of poverty of rural households, particularly focusing on the role of relocation time, reason for relocation, and type of relocation. The results showed that three types of poverty, “voluntary poverty”, “transient poverty”, and “chronic poverty”, are distinguished by combining income and consumption criteria. Moreover, relocation and settlement programs contribute to a certain degree to these three kinds of poverty, and the effects vary according to the relocation characteristics. Specifically, those relocated long-term were more likely to be trapped in “voluntary poverty” and “chronic poverty”, whereas those relocated short-term were less likely to fall into “voluntary poverty” and “transient poverty”. The poverty alleviation and disaster-related resettlers were less likely to be trapped in “chronic poverty”, whereas centralized resettlers were less likely to be trapped in “voluntary poverty” and “chronic poverty”. Additionally, demographic characteristics, capital endowment variables, and geographical features are all important factors affecting rural households’ type of poverty. This study can serve as a reference for further resettlement practice in China and other developing countries.

## 1. Introduction

China has already made widely recognized progress in rural poverty alleviation, but it is now facing lots of new problems and challenges. Not only does the deceleration of rural poverty alleviation contrast with the increasing marginal cost, indicating greater difficulties in lifting the remaining rural poor out of poverty [1], but also the majority of the remaining rural poor are increasingly concentrated in remote and mountainous townships and villages in the western provinces, which are characterized by low educational attainment, poor health, bad living and reproduction conditions, and marginalization [1,2]. Thus, the rural extreme and chronic poor need a development approach and targeted measures for poverty-alleviation policies. A potential policy option for the government is to move people out of these areas, or to transfer people from areas where livelihoods are no longer tenable [3,4]. Some argue that the displacement and resettlement of communities should be a last resort [5]; however, China’s central government has relocated millions of households for several reasons, including infrastructure construction (urban demolition, transportation, and hydropower projects). Recent relocation policies have also seen large numbers of people moved to alleviate poverty, address ecological and disaster-related concerns, and make way for major infrastructure construction projects [6,7,8,9]. Many of those being relocated are moving for the first time in their lives away from their rural ancestral homelands and into newly constructed villages, generally located in the same regions [10]. 

Controversy has arisen and drawn on in relation to past relocation programs in the scholarly literature and news media reports, especially the Three Gorges Project Resettlement (TGPR) [11,12]. This resettlement project was characterized by its involuntary nature and resulted in conflict and violence that was reported in the media. It should be noted that the current conceptualization of relocation as either involuntary or voluntary is too rigid, because the boundary between them is difficult to be strictly distinguished [13]. According to government officials and researchers, the income of resettlers has generally declined and livelihoods were dismantled despite improvements in infrastructure and housing at the TGPR [14].

In response to the TGPR, recent state-organized and policy-directed relocations are expected to be voluntary. The state requires the government at each level to ensure that relocated people are actually willing to move, provide support policies and commercial loans, and encourage them to make their choices about when, where, and how to be relocated. This can be described as continuum of voluntarism or “induced voluntarism” and “compulsory voluntarism”, as suggested by some research [10,13]. One such policy, the Relocation and Settlement Program of Southern Shaanxi Province (RSP, or Program) cannot be conceptualized as primarily a function of resettlers’ decisions, because it reflects the actions of the state and aims of local governmental development planning [15]. Being different from the project-induced relocation, the RSP aims to achieve sustainable levels of development, restore critical ecosystem services locally, regionally, and globally, alleviate poverty and improve rural households’ livelihood security, enhance social welfare, as well as foster rapid economic growth [7,8,9,15,16]. 

Although skepticism and criticism were addressed on the resettlement programs in China, some argue that other developing countries could learn from this model, as the magnitude of the impact (in terms of development improvement) far exceeds that of any other similar project in the world [10,13,17]. As poor rural households have limited access to arable land, instead possessing land of good quality and high production, villages are always located far from the township. Moreover, the carrying capacity of the ecosystem is currently too limited to provide enough resources for the local population, especially considering that the areas are already disaster-prone in the summer, manifested in re-occurring floods and landslides. Thus, not only should government at all levels be expected to improve the conditions of production and living for poor people, share social public resources, block recent population distribution, enhance the self-development ability of the poor, and realize the win-win goal of ecosystem services and human well-being, but also, they should consider poor households eager to change their current bad situations through enhancing their livelihood security and jumping out of the poverty trap. Given the magnitude and informal resources of RSP in western China, understanding how relocation and settlement is likely to influence type of poverty is an issue of considerable significance.

According to research, the resettlement programs were often described as having the potential to create impoverishment based on the social, economic, and mental health of resettlers [11,12,13,18,19]. For instance, using panel data, Hwang et al. [12] showed that relocation had some negative impacts on the economic well-being of the displaced, and many of the negative changes were statistically significant, though the resettlers had access to relatively improved housing stocks. Webber and McDonald [19] found that although the government had the capacity to organize relocation among villages and to provide land and monetary compensation for loss of assets in rural households, the future material well-being of the resettlers was uncertain. Moreover, Scudder [20] argued with no statistical evidence that resettlement outcomes had improved over time. Specifically, in 82% of cases, the living standards of the majority worsened, not just economically, but also socially and culturally [14]. By contrast, some research suggested that relocation projects have access to rural households’ livelihood security, better housing quality, and better public services [13,16]. Xue et al. [13] argued that in many cases the resettled population in the survey region had greatly benefited from relocation. Not only have the relocated retained their productive capacity, but they have also enjoyed roads, markets, school facilities, and other social benefits.

Previous studies contributed significantly to understanding the social-ecological impacts of displacement and resettlement across the globe [3,6,7,8,9,10,13,15,16,21,22,23], especially development-induced displacement and resettlement (DIDR) [11,12,18,19,24,25]. However, most of the studies mentioned above are limited and restricted to environmental issues (water quality and geological instability) and population problems (economic outcomes and health risk). To date, there has been limited research on the relationship between rural households’ poverty and displacement and resettlement. However, unlike in other places where displacement and resettlement is mostly a by-product of large development projects, in China, relocation and settlement has become central to China’s poverty-alleviation practice with Targeted Poverty Alleviation and the goal of ending poverty [9,23]. Teasing out the relationship, the linkage and interaction between relocation and rural households’ poverty might contribute to the body of knowledge and benefit local policymaking toward sustainable development.

Since Cernea [26] proposed the impoverishment risks and reconstruction (IRR) model, which described seven different impoverishment risks through displacement, several scholars followed and employed this empirical framework to reflect negative social impacts for those resettled [27]. Moreover, the definition and classification of poverty have not yet reached agreement. The authors of References [28,29] claimed that poverty could be defined as “transient poverty” and “chronic poverty” from the perspective of poverty dynamics. Further, the capability approach, which was adopted by Sen, suggested that poverty was one type of deprivation on a human’s basic capabilities, rather than just lower income [30]. Therefore, poverty is multi-dimensional, encompassing material deprivation, the lack of access to other basic needs (e.g., education, health, nutrition, and food security), the absence of political autonomy and empowerment, and the lack of freedom of choice and social inequality [31]. This article intends to shed some light on the influence of the RSP on type of poverty by referring to the notion of three types of poverty in urban China [32], because the Chinese government relocated people to developments by constructing high-density resettlement sites in small townships and peri-urban areas [9], which strived to protect the environment and improve quality of life as the EAFRD (European Agricultural Fund for Rural Development)/EFRD (European Fund for Rural Development) did in Europe [33,34].

As mentioned above, research findings on the implication (e.g., social, economic, mental health) of relocation are also limited to project-induced resettlement, which only accounts for a small part of all state-organized relocation. To examine the type of poverty and implications of voluntary relocation in the context of state-organized migration in China, this paper intends to address four research problems: (i) What is the proportion of types of poverty in the survey region? (ii) Does relocation time influence the differences between relocated and non-relocated? (iii) Does the reason for relocation matter, especially poverty-alleviation relocation, and is it different from other reasons for relocation? (iv) Does type of relocation (centralized, scattered, township/urban, self-determined, or other) matter?

## 2. Materials and Methods 

### 2.1. Research Area

**Figure 1:**
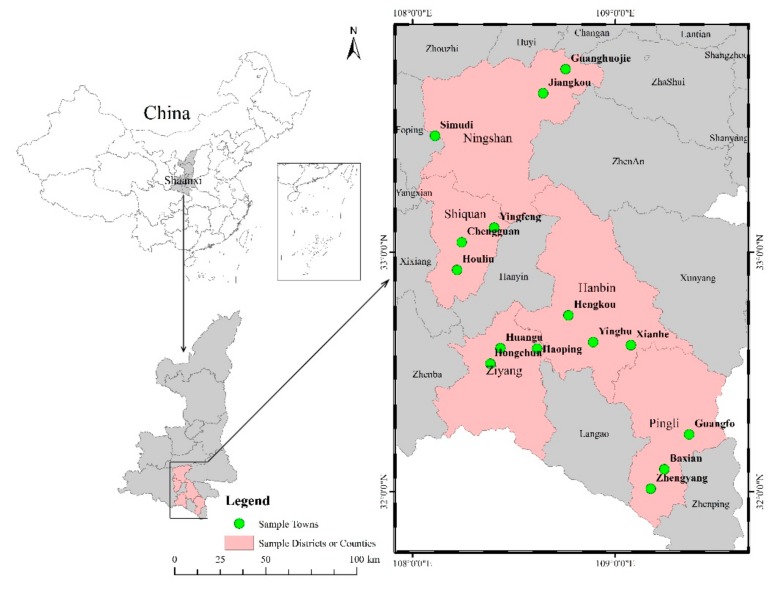
Locations of sample counties (districts) and sample towns.

This study used primary data that our research team collected in Ankang prefecture (an administrative division between the provincial- and county-level), Shaanxi province, China. Ankang prefecture is located in the southern Shaanxi province at the northern base of the Daba Mountains and south of the Qinling Mountains on the upper stream of the Han River. This river is the major tributary of the Yangtze River. Ankang prefecture is also an important water resource conservation area for the South-to-North Water Transfer Project (SNWTP)—the largest water transfer project in the world, designed to deliver high-quality fresh water to arid North China by reducing soil erosion and nutrient runoff into the Han River (see Figure 1 of References [7,16]). With mountains covering most of the region, Ankang prefecture comprises nine counties, together spanning 23,534 km^2^, and all but one county (Pingli) are state-designated as poor or extremely poor. 

Ankang prefecture typifies much of Western China, facing a vexing dilemma between environmental conservation and livelihood improvement, and, together with its two neighboring prefectures, has historically been a disaster-prone region (frequent floods, landslides, debris flow, and so on). From the viewpoint of central and provincial governments, moving persons who live in villages considered to have insufficient carrying capacity or to lack infrastructure and public services, or disaster-affected areas and areas where development is now prohibited for conservation purposes, is an essential instrument apart from poverty alleviation to achieve a diverse set of developmental and environmental objectives. Therefore, Shaanxi province initiated a disaster avoidance and preparedness project in 2011 called RSP—the largest resettlement program in the history of modern China, which was strongly anticipated to facilitate disaster mitigation, accelerate economic development and urbanization, and improve human well-being. Thereafter, with the introduction of Xi Jinping’s Targeted Poverty Alleviation, and the goal to eradicate absolute poverty by 2020, the RSP, now renamed poverty resettlement, has become central to China’s poverty-alleviation practice. According to official statistics, a total of 16 million poor and non-poor people will be resettled in the 13th Five-Year Plan period (2016–2020) [35], which typifies an unprecedented use of relocation and resettlement as poverty-alleviation intervention. Wherein, Shaanxi province aims to resettle 1.25 million people.

This anti-poverty intervention is being implemented at an enormous cost. It contains a 300 billion Chinese Yuan budget allocated to housing alone [35]. The funding for poverty resettlement comprises central and local budget contributions, special development funding, low-cost long-term loans, central government transfers, relocation households’ contributions, and other local government funding. In Shaanxi province, the Implementation Plan for Poverty Resettlement Work has many detailed guidelines and budget rules. Specifically, there is 70,000 Chinese Yuan per poor household capped for housing construction, 50,000 Chinese Yuan per capita capped for infrastructure and basic public facilities (kindergartens, primary schools, hospitals, health clinics, sewage and garbage treatment equipment, and so on). Furthermore, local industries in the host settlement region are subsidized for 30,000 Chinese Yuan, which will be spent on the construction of new agricultural and industrial parks, investment enhancement, and low-interest loans for relocation households to engage in non-farm activities [35]. This subsidized approach intends to establish local specialized industries and to intensify the employment prospects of poor relocation families, with the object of enhancing livelihood diversification. To promote employment in resettlement communities, Shaanxi province offers free training programs and employment guidance, and even prioritizes community public welfare jobs (security, gardening, and maintenance) for those struggling to find employment at the household level, while promoting and subsidizing agricultural or industrial parks and also raising external capital to help the relocated obtain new jobs and positions at the community level [9]. Therefore, all these strategies are utilized to support poor resettlers’ livelihoods for becoming wage labor.

### 2.2. Data Source

The study was based on primary cross-sectional survey data collected in relation to rural households’ livelihoods and the ecological environment in Ankang prefecture [7,10,15]. Our research team at the Institute for Population and Development Studies of Xi’an Jiaotong University conducted the survey using a multi-stage stratified sampling design and structured questionnaires for rural households and communities. Moreover, some semi-structured individual interviews and focus groups were also scheduled. A trained team of investigators collected the survey data with face-to-face interviews held in respondents’ households, and the head of the household, or a family member over 18 years old, was designated to finish the interview. The five focal counties (of nine) in Ankang prefecture were first arranged according to their economic development levels: Hanbin from the first rank (1st), Ziyang, Shiquan, and Pingli from the second rank (4th, 5th, and 6th, respectively), and Ningshan from the third rank (9th) (Figure 1). Then, each focal county was divided into three strata (original villages with non-relocation households, settling villages consisting of all relocation households, and settling villages with mixed relocation and non-relocation households). Thirdly, we selected a random sample of villages from each stratum. Finally, 25 villages were selected, and from the survey villages, villager groups were randomly chosen according to the household registration information from the local statistical bureau. 

A total of 1570 questionnaires were distributed, of which 1410 were validly collected. Wherein, 408 relocation households and 996 non-relocation households were included in the whole sample. The structured household questionnaire focused on the household level, including basic information of households’ social and demographic features, livelihood capitals (natural, financial, social, physical, and human capital), livelihood activities (crop production, forestry planting, local non-farm activities, rural-urban cyclic migration, and so on), consumption and expenditure, and labor time and displacement and resettlement status. 

### 2.3. Variables Selections

#### 2.3.1. Dependent Variable

The poverty line identifies the group in poverty [32]. There is an extensive body of literature on China’s urban and rural poverty [32,36,37,38], and the features and determinants of “transient poverty” and “chronic poverty” in rural China [28,32,39]. However, “voluntary poverty” (a new, or an increasingly important type of poverty) of rural households and its relationship with RSP in the context of Targeted Poverty Alleviation has drawn little attention. Jalan et al. [28] demonstrated that both “chronic” and “transient poverty” were reduced by greater command over physical capital; however, higher variability over time in physical wealth was associated with higher “transient poverty” but lower “chronic poverty”. After analyzing household head age, household size, labor participation, and education, Knight et al. [37] demonstrated that all these factors affected “chronic poverty” more than “transient poverty”, and its impact on consumption poverty was more significant. What contributes to “transient poverty” is extremely occasional, but the cause of “chronic poverty” is difficult to overcome in a short time. Only a few studies have focused on “voluntary poverty”. The authors of References [32,36] considered that poverty in urban China should be divided into “voluntary poverty”, “transient poverty”, and “chronic poverty”. Considering that RSP, as a transition from rural to urban life, is dovetailing with other policy goals to accelerate urbanization [9], and this kind of state-led urbanization is a key part of China’s transition to a consumption-driven economy [40], this study intends to refer to the notion of three poverties in urban China to illuminate the relationship between RSP and poverty. 

Poverty is normally measured in terms of income or consumption in practice, whereas the income-based and consumption-based approaches to poverty have different advantages and disadvantages [32]. Generally, scholars use the notion of permanent income to distinguish between “chronic” and “transient” poverty; however, there is a possible alternative approach to defining poverty. Building on previous studies [32,36] and developing a related theory, we place the poverty type of rural households into three categories: voluntary poverty, transient poverty, and chronic poverty. Corresponding to these three categories, the dependent variable in this article is coded from 1 to 3 for all rural households. Specifically, *Y_i_*= 1 represents “voluntary poverty”, *Y_i_*= 2 represents “transient poverty”, and *Y_i_*= 3 represents “chronic poverty”. Here, we illustrate how they can be defined/distinguished. A household is defined as being in “voluntary poverty” if consumption is below the poverty line but income is above it. Households with an income above the threshold of the minimum poverty line are considered non-poor in this survey. One could also categorize these differently, namely by the rate of consumption. This would, however, alter the classifications for some of the households. Some would become poor instead. A household with consumption above the poverty line but income below it is in “transient poverty”. It can be the case that their predicted income is higher than the actual income or they use savings. A “chronic poverty” household is where both consumption and income are below the poverty line from one period to another. All these three distinctions are different from the chronic/transient disparity. In fact, none of the three concepts corresponds to the precise or loose meaning of chronic or transient poverty. Moreover, the three types of poverty add up to overall poverty. Table 1 describes the dependent variable.

#### 2.3.2. Independent Variables

Following our previous studies and the information obtained from the field survey [7,8,16,38], the independent and control variables in this article involve relocation factors, rural households’ capital endowments (physical capital and financial capital), household demographic traits, and geographical characteristics, which are used to mirror household and geographical factors influencing rural households’ poverty. The descriptive statistics of independent variables and control variables are set out in Table 1. 

This article chooses the categorical variable of "whether relocate or not", "relocation time", "reason for relocation", and "type of relocation" to characterize relocation factors. The variable "whether relocate or not" is a direct reflection of the RSP’s impact on rural households. There are significant distinctions in the context of relocation policies, diversified compensation, and social development environment in the light of relocation time, which determines whether the peasants are poor [8]. The RSP in southern Shaanxi was initiated in 2011, before which numerous resettlement-policy interventions and activities were implemented sporadically, whereas the scale, subsidies, and support were much less than later policies [41]. In particular, there was no economic compensation, multiple preferential policies, or follow-up support for the relocated before 2000. Therefore, the resettlers could be placed into three types according to the time of resettlement: long-term (before 2000); medium-term (2001–2010), and short-term (2011 and after) relocation households. Similarly, here we use a categorical variable to address the reason for relocation, which includes poverty alleviation, ecological restoration, project-induced, disaster-related, and other reasons [10,15,16]. Most resettlers were moved due to infrastructure development (27.45%) and disaster avoidance (26.96%), and comparatively few (12.75%) were moved because of ecological restoration. Moreover, "type of relocation" can accurately characterize the homogeneity of regional geographic features. Its impact on a rural household’s livelihoods varies. Following a previous study [15], the relocated peasants could be divided into five types: centralized, scattered, township/urban, self-determined, and other relocation. About 63% of resettlers were moved through centralized relocation, which involves moving the whole village to a custom-built community. Almost 29% of respondents were scattered into varieties of original communities and 5% made their own choices to determine the settling sites. Among these self-determined relocation households, most of them moved to urban regions or to villages where their relatives lived [15].

#### 2.3.3. Control Variables

Table 1 shows two types of control variables: demographic factors and geographical features. Demographic factors include household head age, demographic characteristics, socioeconomic status, and health. The household head is generally the top decision-maker of a family, whose age can play a vital role in the family’s poverty. For demographic characteristics, two indicators of household size and education are used—both are continuous variables. They can reflect human capital of rural households precisely, which are key factors that affect rural households’ livelihood activities and incomes [38]. Disease is the biggest risk for household poverty, which can seriously affect villagers’ enthusiasm for production and living. Once a family member suffers from illness, sometimes it is a fatal shock to the rural families. Thus, health is used to characterize the illness risk of rural households. According to the authors of references [42,43,44], “whether have out-of-school children” can intuitively influence the off-farm income, thereby further affecting the total income of rural families, as it restrains the supply of off-farm labor. According to the households’ livelihood strategies, rural families can be placed into two types: Pure agricultural households (all labors are engaged in agriculture) and households with combined occupations (the labor force partly employed in agriculture and partly in non-farm activities) [45]. Participation in the sloping land conversion program (SLCP) has significant positive effects on the villagers’ income, particularly for those households on low and medium income; however, participation also has certain negative effects on low- and medium-income households [38]. 

The capital endowment variables mainly include physical capital and financial capital. Here, we use "per capita area of arable land" and "family assets" to characterize physical capital. Wherein, family assets include productive tools, vehicles, and durable goods. All 11 items (assets) have been standardized; the method refers to Liu et al. [8]. Following our previous study, the variable of financial capital is composed of three indicators, "whether the household has loan", "whether the household has deposit", and "whether the household has borrowed money from relatives". The method of capital indicators quantification followed Liu et al. [46]. 

Geographical features concern whether a village is located in an assigned nature reserve or not, and which county the people in the household lived in. If the geographical location of the village is in or adjacent to nature reserves, rural households’ production behavior and livelihood choice tend to be somewhat restricted. Actually, whether there is an adjacent reserve also measures the degree of residents’ access to natural resources, which is an essential factor influencing the diversification of livelihood approaches [38].

### 2.4. Econometric Method

In the present study, the dependent variable of the model, type of poverty, is an unordered multi-classified variable, and can be placed into three categories: voluntary poverty, transient poverty, and chronic poverty. Also, the independent variable and control variable include not only categorical variables (e.g., reason for relocation) but also continuous variables (e.g., per capita area of arable land). Therefore, the multinomial logistic model [47,48] is built to explore the correlation between relocation and settlement and type of poverty. It is noted that employing a logistic model can ensure that each estimated probability lies within the bounds of zero and unity; thus, the relevant probabilities in the present case study sum up to unity in the multinomial logistic model [48]. 

In the regression model, we designate one possibility, non-poor, which denotes the base or reference group. A logarithm of odds (relative to the non-poor households) of each remaining response is supposed to comply with a linear model. The odds ratios are the natural logarithm of the regression coefficients. They have no upper extreme but do have a lower bound of zero. The maximum likelihood estimation (MLE) can be used in lieu of ordinary least squares (OLS) for fitting both the logistic model and the multinomial logit model to estimate the regression parameters. The abbreviated basic regression equation is as follows:

(1) $$\log it(Y)=\alpha +\beta_{1}x_{1}+\beta_{2}x_{2}+\cdot \cdot \cdot +\beta_{i}x_{i}+\varepsilon$$

where *Y* refers to the type of poverty of rural households,α and $\beta_{i}$ represent the assessed parameters of the model, $x_{i}$ refers to the independent variables and control variables, and ε refers to the model residual. The software used was STATA 14.1 (Stata Corp. LLC, College Station, TX, USA).

## 3. Results

### 3.1. Scale of Type of Poverty

Table 1 shows the descriptive statistics for all variables used in the analyses of those who relocated and those who did not. The three types of poverty do not differ significantly: Those who had relocated account for 26.09% (for “chronic poverty”) to 45.34% (for “transient poverty”), compared to 27.67% (for “voluntary poverty”) to 44.23% (for “transient poverty”) for those who had not relocated. Among those who were classified as relocated, the most common period given was medium-term (54.89%), followed by long-term (23.06%) and short-term (22.06%). The lowest number of resettlers were relocated for ecological restoration (12.75%) and poverty alleviation (25.25%), which were almost similar to disaster-related (26.96%), and most people (27.45%) were relocated because of infrastructure projects. For type of relocation, almost 63% of resettlers were relocated via centralized relocation, which involves moving to a new, purpose-built village. About 29% of resettlers were scattered across various pre-existing villages, and 5% made their own decisions about where to relocate.

Relocated and non-relocated individuals differ with regard to some but not all of the control variables. There are no significant differences between the two groups regarding household head age, education, and health. By contrast, those who had relocated were pure agricultural households, SLCP participators, and living in a nature reserve. They had a significantly bigger household size, a higher percentage of out-of-school children, larger family assets and financial capital, but a smaller area of arable land. Finally, there were also significant differences in the regional variable.

As can be seen from Table 2, those identified as poor by both the income-based and consumption-based approaches account for 47.51% of the total survey sample. Among the rest, 27.89% are in "voluntary poverty", 44.52% in "transient poverty", and 27.57% in "chronic poverty". Here, we noticed that the evaluation of poor respondents was higher than that procured by either of the two approaches alone. If we employ the income-based approach to measure poverty, the poverty rate is 34.14%. However, the poverty rate is 26.24% if we utilize the consumption-based approach to estimate poverty.

Table 2 also shows the incidence of the three types of poverty and the overall poverty across the sample areas. There are some differentiations in poverty rates among the five survey counties. For instance, the overall poverty rate of Ziyang county is 5.36%, whereas the incidence of "voluntary poverty" and "chronic poverty" are the lowest in the five counties. Similarly, there is a certain diversification in the incidence of “voluntary poverty” among these five regions. Additionally, the income and consumption of the relocated and non-relocated are also contrasted. The per capita net income of all relocated households is 6705.48 Yuan, and for non-relocated individuals it is 5610.08 Yuan; whereas the relocated households’ per capita consumption is 13,846.77 Yuan, and for non-relocated households it is only 5618.00 Yuan. Correspondingly, the incidence of the three types of poverty and the overall poverty of relocation households is much lower than those of the non-relocated individuals.

Considering relocation time, it is found that long-term relocation households are easily increased to poverty. The poverty incidence of centralized relocated households is lower than scattered individuals, but "transient poverty" incidence is higher than the scattered relocated households. Comparatively speaking, central and local government should take more care of scattered relocated individuals. 

It is demonstrated that the relocated individuals have diverse resilience and poverty type in face of remarkable social development intervention, and this could be their own doing and/or the different characteristics and properties of the external environment that the relocated are faced with. As follows, this study intends to use a multinomial logistic regression model to specifically explore the impact of government-organized relocation on rural households’ type of poverty. 

### 3.2. Regression Results

Table 3 shows the multinomial logistic regression results for the impact of state-organized relocation (resettlement time) on rural households’ poverty type. The detailed estimated results are interpreted as follows. With regard to “voluntary poverty” and “chronic poverty”, the results show that long-term relocation indeed has a positive correlation with these two types of poverty. Conversely, medium-term relocation has a negative correlation with “voluntary poverty” and “chronic poverty”, whereas short-term relocation has a significantly negative correlation with “voluntary poverty” and “transient poverty”. The results show that household head age has a statistically significant positive influence on “voluntary poverty”, but strong negative effect on “transient poverty”, and no effect on “chronic poverty”. Household size has a clear positive influence on the three types of poverty. Education has a significantly negative correlation with “chronic poverty”. The medical care costs accounted for 20%–50% and below 20% have a significantly negative effect on “transient poverty” but no effect on other poverty types. Having out-of-school children in a family has a positive influence on “voluntary poverty”. Sloping land conversion program participation has a clear negative effect on “transient poverty” but no effect on other poverty types. Household type, arable land area, family assets, financial capital, and nature reserve location have a significantly negative correlation with the three types of poverty, but arable land area has no effect on “voluntary poverty”. Being different from other counties, Ningshan county has a clear positive influence on “chronic poverty” but no impact on other poverty types.

Table 4 reports the results of the multinomial logistic regression model assessing the impact of each reason for relocation on poverty type. Here, the results suggested that poverty alleviation relocation appeared to have strong negative correlation with “chronic poverty”, but households who were relocated for poverty alleviation are not significantly different from non-relocated on “voluntary poverty” and “transient poverty”. It is demonstrated that ecological restoration relocation has no significant correlation with the three types of poverty. The results also demonstrated that disaster-related relocation appeared to have a somewhat stronger negative correlation with “chronic poverty” than it did with “voluntary poverty”. The impact of other explanatory variables in the model is extremely similar to that presented in Table 3.

Finally, Table 5 reports the results of the multinomial logistic regression model assessing the impact of each type of relocation on poverty type. In Table 5, the regression compares five different relocation types, controlling for household characteristics, which include household head age, demographic characteristics, socioeconomic status, health, and geographical features. The purpose of this analysis is to observe if certain types of relocation are more harmful than others. The relocation type includes centralized relocation, scattered relocation, personal relocation to townships or urban areas, self-determined relocation, and other forms of relocation. It is demonstrated that centralized relocation has a stronger negative correlation with “voluntary poverty” and “chronic poverty” than it did with “transient poverty”. In fact, it is shown that centralized relocation has no relationship with “transient poverty”. Surprisingly, scattered relocation has a significantly positive impact on “voluntary” and “chronic poverty” and no impact on “transient poverty”. Other forms of relocation have no significant correlation with the three poverty types. The impact of other explanatory variables in the model does not vary much, the significance level and symbol is extremely similar to those presented in Table 3.

## 4. Discussion

Compared to previous studies, the marginal contribution of this article was that the notion of “voluntary poverty” was given to illuminate emerging poverty in the context of RSP, and that the econometric model was employed to disclose the potential mechanisms that allowed rural households to fall into poverty traps from the perspective of rural households’ relocation characteristics, household demographic factors and geographical features. Compared with other studies focusing on project-induced or ecological restoration resettlement, the results of this study may serve as more helpful references in poverty practice and the establishment of new schemes and developmental strategies for the RSP in future phases.

The present study found that most of the changes for the poor fall into the category "transient poverty", whereas those for "voluntary poverty" and "chronic poverty" are quite similar and far more limited. A reasonable explanation might be the predicted income of "transient poverty" poor households greatly exceeded their actual income; however, they also suffered from some form of financial deprivation, either through loss of income sources, inadequate cultivated land allocations, increased living expenditure in new communities, or extensive borrowing to finance new houses [7,8,22,23,49,50,51]. Indeed, by employing the income-based and consumption-based approaches, this study can extend the coverage of poverty, distinguish diverse types of poverty among survey respondents, and also illuminate our comprehension of livelihood choices of the relocated.

Relocation contributes to the three types of poverty, and the effects vary according to relocation time. In essence, earlier migrants’ (especially the project-induced relocated) original livelihood system and social cohesion have been deteriorated; thereby, the loss of capability and yield made them descend into poverty more easily. Although movers who resettled after 2000, especially those who benefited from the mass relocation program, namely the voluntarily relocated, did not fall into “interventional poverty” [26,52]. The “interventional poverty” has its sources, but capability impairments made by resources reconfiguration contribute more to poverty, including loss of assets, employment ability, risk response capability, and human capital accumulation. Meanwhile, disadvantaged households cannot gain excess yields which were brought by their loss of resources [52]. The dual loss of resources and yields leads to relocated households falling into the poverty trap and becoming a vulnerable part of the population. The medium- and short-term relocated chose the independent and rational decision-making behavior of livelihoods, which were based on the high expectation of voluntary resettlement. The relocated usually strive to achieve self-development. However, they still require sufficient time to absorb and buffer the external pressures and shocks. 

The medium-term relocation households were given relatively low subsidies to afford the costs of resettlement and living, and they may also buffer the variation in livelihood approach and reduce their livelihood vulnerability, thereby improving the adaptive capacity and resilience in new settling communities. This may be why the correlation between medium-term relocation and “transient poverty” is not significant. However, the short-term (2011 and after) relocated had contributed a very limited amount to the cost of their new house and living expenditure, some relocated even reporting that their apartment was free to live in. Therefore, debt problems and household contributions, which resulted in extensive borrowing to finance a new apartment and financial deprivation, thereby aggravating “transient poverty”, were effectively controlled. This may be why short-term relocation has a significantly negative impact on “transient poverty”. Further, the correlation between short-term relocation and “chronic poverty” is not significant. This is not surprising. Not only did the short-term relocated require sufficient time to rebuild their livelihood and adapt to the new environment, but also more and more poor people with a dossier were resettled after the implementation of Targeted Poverty Alleviation. Therefore, the government should attach more importance to addressing this subsequent difficulty after resettlement. 

It is noted that centralized relocation has a stronger negative correlation with “voluntary poverty” and “chronic poverty”. This may highlight follow-up supporting policies and measures, such as governmental public service, labor training programs, employment opportunities, and market environmental improvement, which were well implemented in centralized resettlement communities. These policy interventions can help the relocated extend their channels of income. Meanwhile, centralized relocation is definitely convenient for central and local government to plan intensive labor training, employment guidance, and improve the chances of engaging in various non-farm activities, all of which contribute to poverty alleviation. The finding of scattered relocation resulting in poverty was inconsistent with research by the authors of References [8,16]. It may be explained that these relocated individuals were supported by a limited amount of compensation but encountered several forms of financial deprivation or ineffective relocation cost control. Moreover, it is generally insufficient for scattered relocated to rely on their own capabilities to cope with the impoverishment risks and shocks of resettlement intervention; rather, they tend to anticipate central and local government in affording some similar targeted policy assistance and employment guidance as they did in centralized settling communities.

This article provides a novel contribution to previous literature, although there are still some deficiencies in it. Firstly, the selected study site was the Ankang prefecture in southern Shaanxi in Qinba mountain, a contiguous poor area; therefore, it is uncertain whether the conclusions are applicable to other contiguous poor areas and other developing countries. Secondly, the central government has its general guidelines and overlapping motives for anti-poverty relocation and settlement and Targeted Poverty Alleviation, while each province develops more detailed policy guidelines, assistance, budgetary funds and development approaches. Hence, it is also unsure whether the research results are generalizable to poverty resettlement in other provinces, such as Shanxi, Ningxia, Guizhou, Sichuan and Guangxi. Thirdly, the survey data we used in this article were cross-sectional, which could not fully mirror the variation of the relocated and the non-relocated; however, this variation among individuals has an essential impact on those who decide to move to the new houses. Indeed, it may bring endogeneity and selection bias to the research results. Furthermore, poverty is dynamic and easily delivered. The authors did not employ the fixed/random effects model to illuminate the relationships between RSP and type of poverty owing to non-ideal cross-sectional data. A follow-up household survey is being considered to monitor the dynamics of the villagers’ poverty and livelihood in a future study. Finally, this study did not explore the impacts of relocation subsidy, relocation distance, and original resettling region on rural households’ poverty, which could be further addressed. Meanwhile, other detailed resettlement modes, such as nearby model urban sites, cross-regional model rural sites, non-model urban communities, and non-model rural communities, could be further explored in the future. 

## 5. Conclusions

Using survey data from the contiguous poor areas during the implementation of RSP, multinomial logistic model was employed to explore the impact of relocation time, reason for relocation, and type of relocation on rural households’ poverty. The results were as follows:

(1) The relocation time had a significant impact on rural households’ type of poverty. The long-term relocated households were more likely to be trapped in “voluntary poverty” and “chronic poverty”, whereas the short-term relocated households were less likely to fall into “voluntary poverty” and “transient poverty”. When keeping all other variables constant, every unit increase in long-term relocation corresponded to an increase in the odds of “voluntary poverty” and “chronic poverty” by a factor of 3.34 and 3.98, respectively; every unit increase in short-term relocation corresponded to a decrease in the odds of “voluntary poverty” and “transient poverty” by a factor of 0.23 and 0.30, respectively.

(2) The reason for relocation and type of relocation also had a significant impact on rural households’ type of poverty. The poverty alleviation and disaster-related resettlement could decrease “chronic poverty”, whereas disaster-related resettlement could mitigate “voluntary poverty”. In addition, correlations between the reason for relocation and relocation type and “transient poverty” were not significant. Further, the centralized resettlers were less likely to be trapped in “voluntary poverty” and “chronic poverty”, whereas the scattered resettlers were more likely to fall into “voluntary poverty” and “chronic poverty”. When keeping all other variables constant, every unit increase in poverty alleviation and disaster-related resettlement corresponded to a decrease in the odds of “chronic poverty” by factors of 0.10 and 0.21, respectively; every unit increase in disaster-related resettlement corresponded to a decrease in the odds of “voluntary poverty” by a factor of 0.35. Similarly, every unit increase in centralized resettlement corresponded to a decrease in the odds of “voluntary poverty” and “chronic poverty” by factors of 0.48 and 0.10, respectively. However, every unit increase in scattered resettlement corresponded to an increase in the odds of “voluntary poverty” and “chronic poverty” by factors of 2.05 and 2.66, respectively.

(3) Meanwhile, regardless of the model, the demographic characteristics, capital endowment variables, and geographical features are all important factors affecting rural households’ type of poverty. The capital variables, including natural capital, physical capital, human capital, and financial capital, are vital factors affecting farmers’ poverty. The geographical variables are also important impact factors for the individuals.

The results of this study also have essential policy implications. For instance, the scattered relocation households were more likely to fall into “voluntary poverty” and “chronic poverty”. Indeed, the government encouraged and requested households to participate in centralized resettlement, whereas sometimes and often this kind of relocation mode on a central and provincial scale is a hugely expensive exercise, and government budgetary funds cannot be fully covered. Therefore, protecting the scattered resettlers from the negative effects of RSP is essential, especially because they tend to be the vulnerable and neglected population. The government should endeavor to engage in public discourse, learn about and further address scattered relocation households’ needs.

## Figures and Tables

**Table 1:** Definition and descriptive statistics of the variables in the model.

Variable	Definition	Relocated		Not Relocated		*p*-Value
		M ^1^ or %	SD ^2^	M or %	SD	
Dependent variable						
Type of poverty						0.885
Voluntary poverty	Dummy indicator (1 = yes, 0 = otherwise)	28.57%	—	27.67%	—	—
Transient poverty	Dummy indicator (1 = yes, 0 = otherwise)	45.34%	—	44.23%	—	—
Chronic poverty	Dummy indicator (1 = yes, 0 = otherwise)	26.09%	—	28.09%	—	—
Independent variable						
Relocation factors						
Relocation time						
Long-term	Dummy indicator (1 = yes, 0 = otherwise)	23.06%	—	—	—	—
Medium-term	Dummy indicator (1 = yes, 0 = otherwise)	54.89%	—	—	—	—
Short-term	Dummy indicator (1 = yes, 0 = otherwise)	22.06%	—	—	—	—
Reason for relocation						
Poverty alleviation	Dummy indicator (1 = yes, 0 = otherwise)	25.25%	—	—	—	—
Ecological restoration	Dummy indicator (1 = yes, 0 = otherwise)	12.75%	—	—	—	—
Project-induced	Dummy indicator (1 = yes, 0 = otherwise)	27.45%	—	—	—	—
Disaster-related	Dummy indicator (1 = yes, 0 = otherwise)	26.96%	—	—	—	—
Other	Dummy indicator (1 = yes, 0 = otherwise)	7.60%	—	—	—	—
Type of relocation						
Centralized	Dummy indicator (1 = yes, 0 = otherwise)	62.75%	—	—	—	—
Scattered	Dummy indicator (1 = yes, 0 = otherwise)	28.92%	—	—	—	—
Township/urban	Dummy indicator (1 = yes, 0 = otherwise)	0.98%	—	—	—	—
Self-determined	Dummy indicator (1 = yes, 0 = otherwise)	4.66%	—	—	—	—
Other	Dummy indicator (1 = yes, 0 = otherwise)	2.70%	—	—	—	—
Control variable						
Demographic factors						
Household head age	The current age of the head of the household	50.03	13.04	50.68	12.63	0.382
Household size	Total number of people in the household	4.17	1.56	3.45	1.52	0.000
Education	Average years of formal education in the total household	6.33	2.50	6.14	2.89	0.252
Health ^3^						0.256
Over 50%	Reference group	13.58%	—	14.08%	—	—
20%-50%	Dummy indicator (1 = yes, 0 = otherwise)	11.11%	—	14.29%	—	—
Below 20%	Dummy indicator (1 = yes, 0 = otherwise)	75.31%	—	71.64%	—	—
Out-of-school children	Dummy indicator (1 = Children are not in school,0 = otherwise)	11.76%	—	7.83%	—	0.019
Type of household	Dummy indicator (1 = Pure agricultural household, 0 = otherwise)	58.33%	—	50.30%	—	0.006
SLCP ^4^	Dummy indicator (1 = Participate in sloping land conversion program, 0 = otherwise)	85.71%	—	78.20%	—	0.001
Others						
Per capita area of arable land ^5^	Per capita area of arable land owned by the family(mu/person)	1.20	3.17	1.70	2.63	0.003
Family assets	Total assets of the rural household are normalized	0.18	0.10	0.15	0.09	0.000
Financial capital	3 indicators are combined ^6^	0.36	0.26	0.25	0.24	0.000
Geographical features						
In-natural reserve	The village where the rural household is located is in a nature reserve	50.75%	—	34.81%	—	0.000
County	Hanbin = 1, Shiquan = 2, Ningshan = 3, Ziyang = 4, Pingli = 5	2.76	1.49	3.23	1.40	0.000

^1^ M—mean; ^2^ SD—standard deviation; ^3^ The percentage of medical care costs to annual family income; ^4^ SLCP—sloping land conversion program; ^5^ 1 mu = 0.0667 hm^2^; ^6^ 3 indicators including whether the household borrowed from the bank, whether it had savings in the bank and whether the household borrowed from relatives and friends.

**Table 2:** Type of poverty in survey area (%).

Category	Overall Poverty	Voluntary Poverty	Transient Poverty	Chronic Poverty
Total sample	47.51	13.25	21.15	13.10
Hanbin	11.32	3.28	4.54	3.50
Shiquan	9.83	4.10	3.05	2.68
Ningshan	9.01	1.94	3.72	3.35
Ziyang	5.36	0.97	3.95	0.45
Pingli	11.99	2.98	5.88	3.13
Rural households				
Relocated	11.99	3.43	5.44	3.13
Not relocated	35.52	9.83	15.71	9.98
Relocation time				
Long-term	4.80	1.27	1.27	2.25
Medium-term	5.85	1.80	3.22	0.82
Short-term	1.20	0.30	0.90	0.00
Reason for relocation				
Poverty alleviation	2.68	0.89	1.56	0.22
Ecological restoration	0.60	0.15	0.37	0.07
Project-induced	5.36	1.34	1.71	2.31
Disaster-related	2.23	0.45	1.41	0.37
Other	1.12	0.60	0.37	0.15
Type of relocation				
Centralized	5.51	1.56	3.57	0.37
Scattered	5.81	1.64	1.49	2.68
Township/urban	0.07	0.00	0.00	0.07
Self-determined	0.37	0.15	0.22	0.00
Other	0.22	0.07	0.15	0.00

**Table 3:** Multinomial logistic model regression results of relocation time.

Variables	Voluntary Poverty		Transient Poverty		Chronic Poverty	
	Coef.	Exp(β)	Coef.	Exp(β)	Coef.	Exp(β)
Relocation time ^1^						
Long-term	1.21 ***	3.34	0.28	1.33	1.38 ***	3.98
Medium-term	−0.52 *	0.59	−0.14	0.87	−1.27 ***	0.28
Short-term	−1.45 **	0.23	−1.21 ***	0.30	−16.68	0.00
Demographic factors						
Household head Age	0.02 **	1.02	−0.02 **	0.98	−0.01	0.99
Household size	0.37 **	1.45	0.21 ***	1.23	0.69 ***	1.99
Education	0.04	1.05	0.01	1.01	−0.16 ***	0.85
Health ^2^						
20%–50%	14.51	2.00 × 10^6^	−1.36 ***	0.26	−0.41	0.66
Below 20%	16.48	1.44E × 10^7^	−2.27 ***	0.10	−0.07	0.94
Out-of-school children (1 = yes)	0.92 **	2.52	0.17	1.19	−0.07	0.93
Type of household (1 = pure)	−0.88 ***	0.42	−1.72 ***	0.18	−2.67 ***	0.07
SLCP (1 = yes)	0.07	1.08	−0.52 **	0.59	−0.41	0.67
Others						
Per capita area of arable land	−0.02	0.98	−0.45 ***	0.63	−0.63 ***	0.53
Family assets	−8.35 ***	0.00	−2.32 **	0.10	−9.24 ***	0.00
Financial capital	−1.06 **	0.35	−0.82 **	0.44	−0.95 *	0.39
Geographical features						
In-natural reserve (1 = yes)	−1.16 ***	0.31	−1.27 ***	0.28	−1.36 ***	0.26
County ^3^						
Shiquan	−0.32	0.72	−1.12 ***	0.32	−0.44	0.64
Ningshan	0.45	1.56	0.21	1.24	1.32 ***	3.76
Ziyang	−2.24 ***	0.11	−0.69 **	0.50	−2.65 ***	0.07
Pingli	−1.04 ***	0.35	−0.92 ***	0.40	−1.23 ***	0.29
Intercept	−17.38		4.38 ***		2.50 ***	
Sample size			1298			
LR chi^2^			852.03			
Prob > chi^2^			0.0000			
Pseudo R^2^			0.2747			

^1^ The reference category is non-relocated; *, **, and *** refer to *p* < 0.1, *p* < 0.05, and *p* < 0.01, respectively; ^2^ The reference category is medical care costs accounted for over 50% of annual family income; ^3^ The reference category is Hanbin.

**Table 4:** Multinomial logistic model regression results of reason for relocation.

Variables	Voluntary Poverty		Transient Poverty		Chronic Poverty	
	Coef.	Exp(β)	Coef.	Exp(β)	Coef.	Exp(β)
Reason for relocation ^1^						
Poverty alleviation	−0.51	0.60	−0.51	0.60	−2.34 ***	0.10
Ecological restoration	−1.18	0.31	0.20	1.22	−1.54	0.22
Disaster-related	−1.05 **	0.35	−0.42	0.66	−1.56 ***	0.21
Other	0.49	1.63	−0.12	0.89	0.62 *	1.87
Demographic factors						
Household head Age	0.02 **	1.02	−0.02 **	0.98	−0.01	0.99
Household size	0.32 ***	1.37	0.18 ***	1.20	0.64 ***	1.90
Education	0.04	1.04	0.02	1.02	−0.17 ***	0.85
Health ^2^						
20%–50%	14.63	2.26 × 10^6^	−1.33 ***	0.26	−0.26	0.77
Below 20%	16.49	1.45E × 10^7^	−2.28 ***	0.10	−0.07	0.93
Out-of-school children (1=yes)	0.89 **	2.43	0.23	1.25	−0.10	0.90
Type of household (1=pure)	−0.85 ***	0.43	−1.71 ***	0.18	−2.69 ***	0.07
SLCP (1=yes)	0.25	1.28	−0.48 **	0.62	−0.15	0.86
Others						
Per capita area of arable land	−0.02	0.98	−0.47 ***	0.62	−0.61 ***	0.55
Family assets	−7.47 ***	0.00	−2.28 **	0.10	−9.15 ***	0.00
Financial capital	−1.05 **	0.35	−0.82 **	0.44	−1.09 **	0.34
Geographical features						
In-natural reserve (1=yes)	−0.86 ***	0.42	−1.25 ***	0.29	−1.03 ***	0.36
County ^3^						
Shiquan	−0.61 **	0.54	−1.28 ***	0.28	−0.94 ***	0.39
Ningshan	0.08	1.08	0.06	1.06	1.08 **	2.93
Ziyang	−2.42 ***	0.09	−0.85 ***	0.43	−2.92 ***	0.05
Pingli	−1.22 ***	0.30	−1.13 ***	0.32	−1.48 ***	0.23
Intercept	−17.22		4.47 ***		2.70 ***	
Sample size			1307			
LR chi^2^			835.22			
Prob > chi^2^			0.0000			
Pseudo R^2^			0.2677			

^1^ The reference category is non-relocated; *, **, and *** refer to *p* < 0.1, *p* < 0.05, and *p* < 0.01, respectively; ^2^ The reference category is medical care costs accounted for over 50% of annual family income; ^3^ The reference category is Hanbin.

**Table 5:** Multinomial logistic model regression results of type of relocation.

Variables	Voluntary Poverty		Transient Poverty		Chronic Poverty	
	Coef.	Exp(β)	Coef.	Exp(β)	Coef.	Exp(β)
Type of relocation ^1^						
Centralized	−0.74 **	0.48	−0.35	0.71	−2.31 ***	0.10
Scattered	0.72 *	2.05	0.16	1.17	0.98 **	2.66
Township/urban	−17.36	0.00	−17.75	0.00	−1.40	0.25
Self-determined	−0.45	0.64	−1.31	0.27	−16.73	0.00
Other	0.65	1.92	−0.10	0.90	−14.90	0.00
Demographic factors						
Household head Age	0.03 ***	1.03	−0.02 **	0.98	0.00	1.00
Household size	0.31 ***	1.36	0.18 **	1.20	0.59 ***	1.80
Education	0.04	1.05	0.01	1.01	−0.15 ***	0.86
Health ^2^						
20%–50%	14.79	2.65 × 10^6^	−1.36 ***	0.26	−0.55	0.58
Below 20%	16.66	1.72 × 10^7^	−2.31 ***	0.10	−0.22	0.81
Out-of-school children (1 = yes)	0.90 **	2.46	0.20	1.23	−0.02	0.98
Type of household (1 = pure)	−0.83 ***	0.44	−1.71 ***	0.18	−2.61 ***	0.07
SLCP (1 = yes)	0.09	1.09	−0.53 **	0.59	−0.33	0.72
Others						
Per capita area of arable land	−0.04	0.96	−0.47 ***	0.63	−0.65 ***	0.52
Family assets	−6.98 ***	0.00	−2.00 *	0.14	−8.04 ***	0.00
Financial capital	−1.19 ***	0.31	−0.82 **	0.44	−1.13 **	0.32
Geographical features						
In-natural reserve (1 = yes)	−0.99 ***	0.37	−1.27 ***	0.28	−1.18 ***	0.31
County ^3^						
Shiquan	−0.37	0.69	−1.00 ***	0.37	−0.41	0.67
Ningshan	0.14	1.15	0.16	1.17	1.07 **	2.92
Ziyang	−2.35 ***	0.10	−0.78 **	0.46	−2.72 ***	0.07
Pingli	−1.13 ***	0.32	−1.05 ***	0.35	−1.23 ***	0.29
Intercept	−17.58		4.48 ***		2.39 ***	
Sample size			1307			
LR chi2			850.64			
Prob > chi2			0.0000			
Pseudo R^2^			0.2726			

^1^ The reference category is non-relocated; *, **, and *** refer to *p* < 0.1, *p* < 0.05, and *p* < 0.01, respectively; ^2^ The reference category is medical care costs accounted for over 50% of annual family income; ^3^ The reference category is Hanbin.

## References

[1] Wang D.W., Cai F. (2006). Migration and poverty alleviation in China. China Labor. Econ..

[2] Cai F., Du Y. (2006). The changing nature of rural poverty and new policy orientations. China Econ..

[3] Tan Y., Zuo A., Hugo G. (2013). Environment-related resettlement in China: A case study of the Ganzi Tibetan autonomous prefecture in Sichuan province. Asia Pac. Migr. J..

[4] Warner K., Hamza M., Oliver-Smith A., Renaud F., Julca A. (2010). Climate change, environmental degradation and migration. Nat. Hazards.

[5] Barnett J., Webber M. (2010). Accommodating Migration to Promote Adaptation to Climate Change.

[6] Guo X.S., Kapucu N. (2017). Examining livelihood risk perceptions in disaster resettlement. Disaster Prev. Manag..

[7] Li C., Li S.Z., Feldman M.W., Li J., Zheng H., Daily G.C. (2017). The impact on rural livelihoods and ecosystem services of a major relocation and settlement program: A case in Shaanxi, China. Ambio.

[8] Liu W., Xu J., Li J. (2018). The influence of poverty alleviation resettlement on rural household livelihood vulnerability in the western mountainous areas, China. Sustainability.

[9] Rogers S., Li J., Lo K., Guo H., Li C. (2019). Moving millions to eliminate poverty: China’s rapidly evolving practice of poverty resettlement. Dev. Policy Rev..

[10] Wu Z., Penning M.J., Zeng W.H., Li S.Z., Chappell N.L. (2015). Relocation and social support among older adults in rural china. J. Geron. Ser. B.

[11] Wilmsen B., Webber M., Duan Y.F. (2011). Development for whom? Rural to urban resettlement at the Three Gorges Dam, China. Asian Stud. Rev..

[12] Hwang S.S., Cao Y., Xi J. (2011). The short-term impact of involuntary migration in China’s Three Gorges: A prospective study. Soc. Indic. Res..

[13] Xue L., Wang M.Y., Xue T. (2013). ‘Voluntary’ poverty alleviation resettlement in China. Dev. Chang..

[14] Wilmsen B., Wang M.Y. (2015). Voluntary and involuntary resettlement in china: A false dichotomy?. Dev. Pract..

[15] Zeng W.H., Wu Z., Schimmele C.M., Li S.Z. (2015). Mass relocation and depression among seniors in China. Res. Aging.

[16] Li C., Zheng H., Li S.Z., Chen X.S., Li J., Zeng W.H., Liang Y.C., Polasky S., Feldman M.W., Ruchelshaus M. (2015). Impacts of conservation and human development policy across stakeholders and scales. Proc. Natl. Acad. Sci. USA.

[17] Rogers S., Wang M.Y. (2006). Environmental resettlement and social dis/re-articulation in Inner Mongolia, China. Popult. Environ..

[18] Webber M.J. (2012). Making Capitalism in Rural China.

[19] Webber M.J., McDonald B. (2004). Involuntary resettlement, production and income: Evidence from Xiaolangdi, PRC. World Dev..

[20] Scudder T., Tortajada C., Altinbilek D., Biswas A. (2012). Resettlement outcomes of large dams. Impacts of Large Dams: A Global Assessment.

[21] Chen Y., Tan Y., Luo Y. (2017). Post-disaster resettlement and livelihood vulnerability in rural China. Disaster Prev. Manag..

[22] Lo K., Xue L.Y., Wang M. (2016). Spatial restructuring through poverty alleviation resettlement in rural China. J. Rural Stud..

[23] Lo K., Wang M. (2018). How voluntary is poverty alleviation resettlement in China?. Habitat Int..

[24] Wilmsen B. (2011). Progress, problems, and prospects of dam-induced displacement and resettlement in china. China Inform..

[25] Hwang S.S., Xi J., Cao Y., Feng X.T., Qiao X. (2007). Anticipation of migration and psychological stress and the three gorges dam project, china. Soc. Sci. Med..

[26] Cernea M.M. (2000). Risks, safeguards and reconstruction: A model for population displacement and resettlement. Econ. Polit. Wkly..

[27] Kirchherr J., Charles K. (2016). The social impacts of dams: A new framework for scholarly analysis. Environ. Impact Assess. Rev..

[28] Jalan J., Ravallion M. (2000). Is transient poverty different? Evidence for rural China. J. Dev. Stud..

[29] Duclos J.Y., Araar A., Giles J. (2010). Chronic and transient poverty: Measurement and estimation, with evidence from China. J. Dev. Econ..

[30] Gasper D. (2002). Is Sen’s capability approach an adequate basis for considering human development?. Rev. Polit. Econ..

[31] Roe D., Walpole M., Elliot J. (2010). Linking biodiversity conservation and poverty reduction: What, where and how?. Biodiversity.

[32] Knight J., Li S. (2006). Three poverties in urban China. Rev. Dev. Econ..

[33] Alonso G.C., Masot A.N. (2017). Towards rural sustainable development? Contributions of the EAFRD 2007–2013 in low demographic density territories: The case of Extremadura (SW Spain). Sustainability.

[34] Masot A.N., Alonso G.C. (2018). The rural development policy in Extremadura (SW Spain): Spatial location analysis of leader projects. ISPRS Int. J. Geo-Inf..

[35] National Development and Reform Commission National “13th Five Year Plan” Poverty Resettlement Plan. http://www.ndrc.gov.cn/zcfb/zcfbtz/201610/t20161031824886.html.

[36] Li S., Knight J. (2002). Three poverties in urban China. Econ. Res. J..

[37] Knight J., Li S., Deng Q.H. (2009). Education and the poverty trap in rural China: Setting the Trap. Oxf. Dev. Stud..

[38] Li J., Feldman M.W., Li S.Z., Daily G.C. (2011). Rural household income and inequality under the sloping land conversion program in western china. Proc. Natl. Acad. Sci. USA.

[39] Knight J., Li S., Deng Q.H. (2010). Education and the poverty trap in rural China: Closing the Trap. Oxf. Dev. Stud..

[40] Wilmsen B. (2018). Damming china’s rivers to expand its cities: The urban livelihoods of rural people displaced by the three gorges dam. Urban Geogr..

[41] Li J. (2016). Livelihood adaptation strategy and perceived adaptive capacity of rural relocated households in 735 Southern Shaanxi Province, China. China Popul. Res. Environ..

[42] Xu D.D., Liu E.L., Wang X.X., Tang H., Liu S.Q. (2018). Rural households’ livelihood capital, risk perception, and willingness to purchase earthquake disaster insurance: Evidence form Southwestern China. Int. J. Environ. Res. Public Health.

[43] Huang L., Huang J., Wang W. (2018). The sustainable development assessment of reservoir resettlement based on a bp neural network. Int. J. Environ. Res. Public Health.

[44] Gichunge C., Somerset S., Harris N. (2016). Using a household food inventory to assess the availability of traditional vegetables among resettled African refugees. Int. J. Environ. Res. Public Health.

[45] Liang Y.C., Li S.Z., Feldman M.W., Daily G.C. (2012). Does household composition matter? the impact of the grain for green program on rural livelihoods in china. Ecol. Econ..

[46] Liu W., Xu J., Li J. (2018). Livelihood vulnerability of rural households under poverty alleviation relocation in Southern Shaanxi, China. Res. Sci..

[47] Xu D.D., Guo S.L., Xie F.T., Liu S.Q., Cao S. (2017). The impact of rural laborer migration and household structure on household land use arrangements in mountainous areas of Sichuan Province, China. Habitat Int..

[48] Xu D.D., Zhang J.F., Xie F.T., Liu S.Q., Cao M.T., Liu E.L. (2015). Influential factors in employment location selection based on “push-pull” migration theory—A case study in Three Gorges Reservoir area in China. J. Mt. Sci..

[49] Fu H.L., Manogaran G., Wu K., Cao M., Jiang S., Yang A.M. (2019). Intelligent decision-making of online shopping behavior based on internet of things. Int. J. Info Mgt.

[50] Jiang W., Carter D.R., Fu H.L., Jacobson M.G., Zipp K.Y., Jin J., Yang L. (2019). The impact of the biomass crop assistance program on the United States forest products market: An application of the global forest products model. Forests.

[51] Rogers S., Xue T. (2015). Resettlement and climate change vulnerability: Evidence from rural china. Glob. Environ. Chang..

[52] Yan D.C., Shi G.Q., Zhou J. (2011). Review of the cause of reservoir relocation poverty under the paradigm perspective. Water Resour. Dev. Res..

